# Chemokines and their receptors: predictors of the therapeutic potential of mesenchymal stromal cells

**DOI:** 10.1186/s12967-021-02822-5

**Published:** 2021-04-17

**Authors:** Nerea Cuesta-Gomez, Gerard J. Graham, John D. M. Campbell

**Affiliations:** 1 Chemokine Research Group, Institute of Infection, Immunity and Inflammation, University of Glasgow, Glasgow, UK; 2 Tissues, Cells and Advanced Therapeutics, Scottish National Blood Transfusion Service, The Jack Copland Centre, Research Avenue North, Edinburgh, UK

**Keywords:** Mesenchymal stromal cell, Chemokine, Chemokine receptor, Tissue source, Therapeutic potential

## Abstract

Multipotent mesenchymal stromal cells (MSCs) are promising cellular therapeutics for the treatment of inflammatory and degenerative disorders due to their anti-inflammatory, immunomodulatory and regenerative potentials. MSCs can be sourced from a variety of tissues within the body, but bone marrow is the most frequently used starting material for clinical use. The chemokine family contains many regulators of inflammation, cellular function and cellular migration–all critical factors in understanding the potential potency of a novel cellular therapeutic. In this review, we focus on expression of chemokine receptors and chemokine ligands by MSCs isolated from different tissues. We discuss the differential migratory, angiogenetic and immunomodulatory potential to understand the role that tissue source of MSC may play within a clinical context. Furthermore, this is strongly associated with leukocyte recruitment, immunomodulatory potential and T cell inhibition potential and we hypothesize that chemokine profiling can be used to predict the in vivo therapeutic potential of MSCs isolated from new sources and compare them to BM MSCs.

## Background

Mesenchymal Stromal Cells (MSCs) are a non-hematopoietic multipotent adult progenitor population that were first named in 1976 by Alexander Friedenstein as colony-forming unit fibroblasts (CFU-Fs). These cells were spindle shaped, clonogenic in monolayer cultures and could serve as feeders in the bone marrow for hematopoietic stem cells [1, 2]. The term “marrow stromal stem cell” was first used by Maureen Owen in 1988 because of their ability to self-renew (although this may be interpreted today as a high proliferative capacity) and the gene activation potential to differentiate into discrete connective tissue cells [3]. Although MSCs were first isolated from bone marrow, they can be isolated from a wide range of tissues around the body, including umbilical cord, cord blood, placenta, dental pulp, periodontal ligament, adipose tissue and pancreatic islets [4]. MSC are now commonly referred to as Mesenchymal Stromal Cells [5, 6] rather than stem cells.

MSCs exert potent anti-inflammatory, immunoregulatory and pro-angiogenic effects through interactions with the immune system and the secretion of paracrine immunomodulators. These characteristics, coupled with their ease of accessibility and expansion resulted in a growing interest in the therapeutic potential of these cells. However, the variety of tissue sources, coupled with different isolation and culture protocols and the intrinsic variability of MSCs from donor to donor has led to wide variability regarding the description of MSC phenotypes and properties through the literature. To address this matter, the International Society of Cellular Therapy (ISCT) specified the criteria human cells had to reach in order to be defined as MSCs: plastic-adherence when maintained in standard culture conditions and specific surface antigen expression along with trilineage differentiation potential into osteoblasts, adipocytes and chondroblasts [5, 6]. MSCs must express CD105, CD73 and CD90; must lack the expression of hematopoietic antigens such as CD45, CD34, CD14 or CD11b, CD79α or CD19 and MHC Class II [6]. MSCs express MHC Class II upon stimulation with IFN-γ; under these circumstances, cells are still termed MSCs but must be qualified with adjectives as ‘activated’, ‘licensed’ or ‘stimulated’ to explain that these cells are not in a resting state. MSCs also express variable levels of other markers including CD29, CD44, CD166, CD146 and CD271, which can allow the isolation of subsets of tissue specific MSCs; CD271, for example, allows the isolation of subpopulations of MSCs associated with bone surfaces and with increased osteogenic differentiation potential and enhanced cartilage repair potential [7, 8].

These criteria have helped to drive standardization in the manufacturing and identity of MSC for therapeutic use, but does not capture whether MSCs derived and cultured from different tissues have equivalent therapeutic potential or potency. The chemokine family contains many regulators of inflammation, cellular function and cellular migration–all critical aspects in designing a novel cellular therapeutic and understanding potential potency. In this review, we focus on expression of chemokine receptors and chemokine ligands by MSCs isolated from different tissues.

## Use of MSCs in the clinic

The use of MSCs as cellular therapeutics is limited by the low frequency of these cells within tissues and the high doses required for medical use. The American Code of Federal Regulation of the Food and Drug Administration and the European Medicines Agency have established guidelines, generally known as “Good Manufacturing Practice” (GMP), that cover cell culture procedures, reproducibility, efficiency, and safety. Most isolation and cell culture protocols are not optimal for GMP adaption, which has limited the tissue sources used for MSC isolation driving clinical use into narrow corridors [9]. According to the ISCT, most facilities involved in MSC manufacturing isolate MSCs from a single source, more precisely, 93.3% of the facilities isolate MSCs from bone marrow, 26.7% from adipose or umbilical cord tissue, 13.3% used umbilical cord blood and 6.7% used placental tissue as a source [10].

It is therefore well established that MSCs can be isolated from most tissues within the body and that tissue source of origin as well as the conditions used to expand the cells influence MSC secretome, and thus, their therapeutic potential [11–13]. For this reason, it is essential to understand the differences between MSCs isolated from different tissue sources to predict in vivo behavior and widen the use of MSCs in the clinic.

### Tissue replacement and inflammatory modulation

Due to their differentiation potential, the initial clinical use of MSC was aimed at tissue reconstruction or regeneration including musculoskeletal tissues, nervous system, liver and skin among others. However, this regeneration potential has only been clinically proven to be effective in MSC-based bone regeneration [14]. Even in this field, while all MSCs have osteogenic differentiation potential in vitro, comparative studies evaluating this ability among MSCs isolated from different sources to regenerate bone are inconclusive. It is difficult to definitively separate the tissue-building capacity of MSC from the ability to modulate inflammatory responses–actively re-building a tissue versus stopping inflammatory destruction of tissue. Tissue regeneration may also be indirect as MSC have pro-angiogenic capacity. Increased angiogenic potential, which is variable among tissue sources of isolation, promotes tissue reconstruction and thus, tissue source of MSC isolation might play an essential role within a clinical setting [12, 15].

In response to their microenvironment, MSCs modulate innate and adaptive immune responses [12] as well as enhance angiogenesis via cell contact interaction and paracrine effects [13, 15, 17]. MSCs secrete a plethora of angiogenic factors including vascular endothelial growth factor (VEGF), hepatocyte growth factor (HGF), transforming growth factor-beta (TGF-β), matrix metalloproteinases (MMP) and chemokines, which stimulate angiogenesis in vitro and in vivo [16–18]. Furthermore, the secretion of anti-inflammatory and immunomodulatory molecules, including interleukin 10 (IL-10), tumor necrosis factor-inducible gene 6 protein (TSG6), TGF-β, indoleamine 2, 3-dioxgenase (IDO) and CD274, avoids effector T cell proliferation and promotes a regulatory phenotype of leukocytes [19–23].

The mechanisms of migration of MSC to defined in vivo niches following systemic administration and persistence in these sites whether by homing or local administration remains largely unexplored. MSC migration and homing is hypothesized to be similar to leukocyte migration from the bloodstream and to involve adhesion molecules for rolling and trans-endothelial migration [24, 25]; chemokines, cytokines and their receptors for chemotaxis [26, 27]; and matrix metalloproteinases for invasion [28].

## Chemokines and chemokine receptors: chemotaxis

**Fig. 1:**
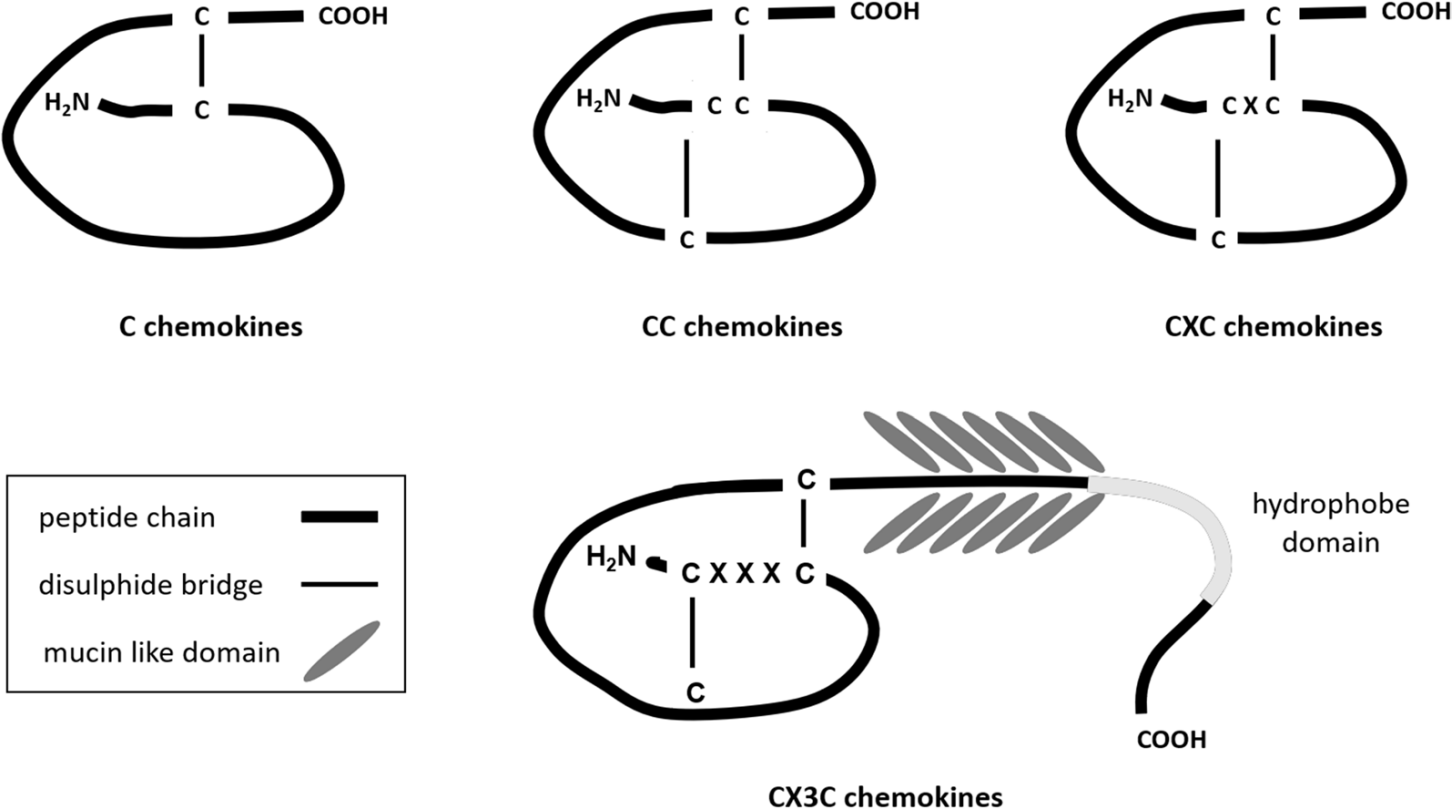
Highly conserved molecular signature of the chemokine subfamilies. Chemokines are classified into 4 families according to the cysteine residues close to the amino terminus of the protein and the disulphide bonds originated due to these residues

Chemokines are a family of small heparin-binding homologous proteins involved in the regulation of cell migration under both inflammatory and physiological conditions [29]. Chemokines are classified according to the presence of a conserved tetra cysteine motif. The relative position of the N-terminal first two consensus cysteine residues provides the basis for their classification [30, 31] (Fig. 1). XCL1 and XCL2 have a single cysteine residue near the amino terminus that enables the generation of a disulphide bond (A), while CC chemokines have two consecutive cysteine residues in the amino terminal (B) and CXC chemokines have two cysteine residues separated by only one non-conserved amino acid residue “X” (C). Fractalkine (CX3CL1), the only known member of the CX3C chemokine family, has two cysteine residues separated by three amino acid residues “X” (D). CX3CL1 and CXCL16 contain a mucin-like domain linked to a hydrophobic, and therefore transmembrane, domain and an intracellular tail that allows them to be presented as cell surface bound chemokines. However, these chemokines can also be found in soluble forms too.

Chemokine nomenclature is based on their structural classification but can also be sorted out into functional categories; homeostatic, proinflammatory and multifunctional according to the microenvironment. Inflammatory chemokines become upregulated in the presence of inflammation, promoting the recruitment of leukocytes. As an example, all nucleated cells can upregulate the expression of CCL2 to induce the recruitment of leukocytes towards a site of injury or infection. Inflammatory chemokines and their receptors display complex interaction patterns; a single ligand has broad receptor selectivity and a single receptor has a broad number of agonists to enable a rapid immune response to protect the host [32]. Chemokines arose from gene duplication from an ancestral chemokine gene which was probably CXCL12, explaining why the most important inflammatory CC and CXC chemokines are clustered in chromosome 17 and chromosome 4, respectively, in humans [33].

The expression of homeostatic chemokines is constitutive and cell or tissue specific [28]. These chemokines are involved in the regulation of cells involved in acquired immunity to ensure proper tissue maintenance and development [29]. CXCL12 is a useful exemplar as it regulates the migration of hematopoietic stem cells during embryonic development as well as regulating lymphocytic circulation to promote immune surveillance post-natally [34]. Homeostatic chemokines are less promiscuous and in many cases have restrictive ligand-receptor relationships, like CXCL12 and its receptor, CXCR4 [29].

Cell migration is induced upon interaction of chemokine ligands with their cognate receptors. Chemokine receptors are part of the γ subfamily rhodopsin-like seven-transmembrane receptors and, unlike their ligands, are well conserved among species. Chemokine receptors can be classified into G protein-coupled or classical chemokine receptors and atypical chemokine receptors (ACKRs) according to their dependency on G proteins for signaling and the atypical expression patterns [35]. Classical chemokine receptors have a conserved DRYLAIV motif (D: Aspartate; R: Arginine; Y: Tyrosine; L: Leucine, A: Alanine; I: Isoleucine; V: Valine), located on the second intracellular loop, which upon interaction with G proteins, enables the production of a calcium flux following the chemokine ligand-chemokine receptor interaction [36].

## Chemokine mediated homing of MSCs

MSCs reside in specific tissue niches where their microenvironment regulates the balance of proliferation and differentiation, as well as their secretome, which includes cytokines, chemokines, immunomodulatory molecules, and growth factors [37]. In this manner, upon tissue injury or inflammation, MSCs can regulate immune responses and promote tissue regeneration to maintain homeostasis. As examples, synovium-derived MSCs and BM MSCs have the potential to promote self-repair of the articular cartilage upon injury [38–40]; while BM MSCs can home to cutaneous wounds where they transdifferentiate into multiple skin cell types and promote cell migration, angiogenesis, epithelialization and collagen production to assist regenerative wound healing [41–43].

Inflammatory response to injury results in the secretion of an array of chemokines in a temporally and spatially orchestrated manner that results in cell migration. The secretion of CXCL8 soon after injury results in the recruitment of neutrophils to the damaged or infected tissue. Neutrophils promote the recruitment of monocytes via the release of CCL2, while the secretion of CCL3, CCL4 and CCL5 by macrophages results in B and T cell migration. MSCs express and respond to chemokines and to date, the CXCL12-CXCR4 axis is the best described axis in MSC homing to wounds.

### The CXCL12-CXCR4 axis

CXCL12 is a homeostatic chemokine and its cognate receptor is CXCR4. ACKR3 is also able to interact with CXCL12 but this interaction does not result in chemotaxis but it has been described to be important for endothelial cell proliferation [44, 45]. CXCL12 or CXCR4 gene knockout in mice results in perinatal death due to defective cardiac ventricular septa, embryonic hematopoiesis and neurogenesis due to the role of CXCL12 in stem cell, endothelial cells and leukocyte recruitment [46, 47]. This axis is not tissue specific and enables the migration of MSCs towards sites of injury in any tissue including brain, heart, skeletal muscle, kidney, liver and skin among others. Gain and loss of function experiments modulating the expression of CXCL12 secretion at the wound or CXCR4 expression by MSCs has demonstrated the role of MSC migration in wound healing. Overexpression of CXCL12 in diabetic and non-diabetic skin wounds accelerated re-epithelialization and wound healing [48, 49]; while inhibition of CXCL12 reduces MSC migration and delays wound healing [50]. Similarly, blockade of CXCR4 on infused MSCs inhibits MSC recruitment towards the injured tissue and retards wound healing [50], while overexpression of CXCR4 increased homing to wounds [51].

More importantly, pre-treatment of MSCs with CXCL12 increases their survival and proliferation potential, as well as enhancing their migration towards injured tissue and increasing the secretion of basic fibroblast growth factor and VEGF in vitro [52]. Moreover, MSCs themselves secrete CXCL12 and thus, promote cell migration and angiogenesis. In fact, transduction of MSCs with a lentiviral vector expressing shRNA against CXCL12 resulted in reduced vascularization, re-epithelialization and wound healing compared to control MSCs [53], showing the essential roles chemokines play in the therapeutic potential of MSCs. MSCs isolated from different tissues have a differential chemokine profile and secretion can be regulated by MSC pre-treatment. Therefore, it is essential to fully understand the role of tissue of origin and pre-treatment in the expression and secretion of chemokines as it could be a potent indicator of the outcome and efficacy of MSC therapy.

From the perspective of transplant clinicians or researchers, pre-treatment of cells and tissues is considered more than minimal manipulation and for commercial use, it requires GMP methodologies to ensure that cells meet the requirements for safety, while maintaining the identity, quality and purity characteristics that the cells are represented to possess prior to ex vivo manipulation [54]. For this aim, the laboratory and the protocols themselves must undergo a process of extensive validation to ensure adequate control of the aseptic processing as well as product and lot-release purity and potency criteria to be released for clinical use [54].

### Other chemokine mediated migration axes

MSCs express variable levels of chemokine ligands and receptors but migration of MSCs towards chemokines has only been described in a few cases.

#### CCL27-CCR10 axis

The CCL27-CCR10 axis has been shown to recruit CD34 + bone marrow derived multipotent cells towards the skin. CCR10 expression has been described in human and murine MSCs isolated from different sources [55, 56] and overexpression of CCR10 in MSCs has demonstrated that the CCL27-CCR10 axis results in MSC migration towards the skin [57]. Administration of a mixture of WT and CCR10 overexpressing MSCs into the dermis of mice and intradermal injection of CCL27 at a site distal from administration resulted in progressive migration of CCR10 + MSCs towards CCL27, while WT MSCs remained at the administration site. Intravenous administration of CCR10 + MSCs and intradermal injection of CCL27 resulted in increased migration of MSCs towards the injection site of CCL27 when compared to mice administered with WT MSCs [57].

#### CCL19/CCL21-CCR7 axis

CCR7 expression is widely described in MSCs and CCL19 and CCL21 are the only ligands for CCR7. High expression of CCL21 by secondary lymphoid organs has been described to be responsible for the non-specific distribution of infused MSCs [58]. However, intradermal injection of CCL21 into wounded mouse skin resulted in increased migration of the intravenously administered MSCs towards the injured site and accelerated wound repair compared to control, where MSC migration was not induced by CCL21 injection [41].

#### CCL5-CCR5 axis

The CCL5-CCR5 axis is involved in the recruitment to wounds of endothelial progenitor cells in vivo [59] and hematopoietic stem cells in vitro [60]. This axis is important in MSC mediated tumor invasion and metastasis due to the increased secretion of the inflammatory chemokine CCL5 by MSCs [61, 62]. However, the role of CCR5 expressed by MSCs remains unclear within a migratory context.

## Chemokine receptors and enhanced therapeutic potential of MSCs

Within a clinical context, the optimal delivery method of MSCs should provide the highest regenerative benefit with the lowest side effects. The most used routes of MSC administration, outside tissue-engineering-based methods, are direct injection into the tissue of interest and systemic infusion, both intra-venously (IV) or intra-arterially. Direct injection should have the advantage of a much more precise localization of the cells; however, only 1 to 5% of delivered cells engraft within the target site regardless of the delivery route [63]. Unlike hematopoietic stem cells, where engraftment and survival of CD34 + cells is essential for long-term and overall therapeutic effect, it is not clear whether MSCs require to engraft and persist to exert their therapeutic roles [64].

MSCs exert their mechanism via the secretion of paracrine factors that interact with surrounding immune and stromal cells resulting in the generation of pro-tolerogenic, pro-regenerative and anti-inflammatory environments and thus, it is not clear if increased engraftment would result in increased therapeutic potential. Systemic infusion is much less invasive and enables easy access to oxygen and nutrients, which is why it is the preferred method for MSC delivery [65]. Systemic infusion of MSCs leads to engraftment of MSCs mostly in the lungs. In the presence of inflammation, MSCs can also be found in the spleen, liver, bone marrow, thymus, kidney and skin, seconds or minutes after IV injection, suggesting that chemotactic agents could be guiding infused MSCs specifically towards these organs [66].

Chemokines are master regulators in immune cell trafficking under resting and inflammatory conditions. Specific chemokines have been shown attract immune cells expressing the cognate receptor to specific tissues: the expression of CCR1 in macrophages and neutrophils leads to kidney infiltration in renal ischemia–reperfusion injury [67]; CCR5 directs CD8 + T cells towards the brain [68]; CCR3, CCR4 and CCR10 are highly expressed by T cells in skin [69, 70]; CXCR3 mediates T cell recruitment into the kidney [71]; and CXCR6 is highly expressed by liver-infiltrating CD8 + T cells [72]. Furthermore, CCR7 targets MSCs to secondary lymphoid organs [73, 74], CXCR3-deficient MSCs fail to infiltrate into the nephritic kidney [75] and CXCR4 receptor overexpression in MSCs improves treatment of acute lung injury [76]. Thus, specific chemokine receptors could have an essential role in MSC homing into specific tissues.

Human and murine MSCs have been described to constitutively express chemokines and their receptors, but the expression of these markers varies among reports in the literature due to tissue source of the cells, cell culture methods and passage number of the cells. Murine BM MSCs have been described to express CCR1, CCR2, CCR3, CCR4, CCR5, CCR6, CCR7, CCR9, CCR10, CXCR3, CXCR4 and CXCR7 and to have functional CCR3, CCR4, CCR5, CCR7, CCR10 and CXCR4 [56]. Adipose derived (Ad) MSCs have higher CXCR4 expression and migration capacity than bone marrow derived (BM) MSCs. More interestingly, the chemokine receptor profile is sensitive to time in culture as the expression of chemokine receptors CCR1, CCR7, CXCR1, CXCR2, CXCR4 and CX3CR1 was decreased after passage [27]. In addition, expression of chemokine receptors and ligands can be upregulated by cytokine-mediated stimulation [77]. All this together suggests that the tissue source of origin of MSCs is associated with differential chemokine receptor expression and therefore, different homing potential to sites of inflammation, which could be of relevance when used as cellular therapeutics.

## MSC engineering to enhance MSC migration

There are several strategies to increase the expression of chemokine receptors and improve MSCs homing efficiency, including genetic modifications and surface engineering. Viral transduction and mRNA transfection result in permanent or transient overexpression, respectively, of specific chemokine receptors. Viral transduction of CXCR4 increases homing to the bone marrow in irradiated mice [78] and enhances homing and recovery of ischemic myocardium [79] and damaged intestinal mucosa [80]. Transient overexpression of CXCR4 has resulted in contradictory results, where Ryser et al., showed increased migration in transwell assays while Wiehe et al., demonstrated functionality of the overexpressed CXCR4 without improvement in cell migration [81, 82]. ACKR3 permanent overexpression improves migration and recovery in a rat model of acute lung injury [83]. Interestingly, ACKR3 overexpression results in a positive feedback loop where MSCs increase the expression of CXCL12, vascular cell adhesion protein 1 and the surface adhesion receptor CD44, further enhancing migration [84]. Similarly, CCR2 transduction increases migration to and retention within ischemic brain lesions and improved the neurological outcomes in an ischemic stroke rat model [85]. Cell surface engineering focuses on transiently modifying or conjugating desired ligands to existing surface proteins. Won et al., were able to conjugate recombinant CXCR4 to MSC membranes, improving migration towards a CXCL12 gradient in vitro [86].

While cell engineering helps improve understanding of the roles of different molecules expressed or secreted by MSCs, the design of good manufacturing practices for cell-engineering is challenging. For cell engineering, large quantities of concentrated vectors must be produced, and cells must be transduced in different areas to avoid contaminating the transduction area with concentrated vector from the production rooms. If the facility produces multiple vectors, separate aseptic rooms for vector production are essential, as well as stringent decontamination processes and scrutinized personnel flow and use of shared equipment to avoid contaminating different areas within the facility. Cell transduction itself, presents different risks of cross-contamination regardless of the smaller quantities of vector required. If different vectors are being transduced for clinical trials or commercial use, it is essential to establish process control systems to ensure vector isolation when in use of common equipment. Cell engineering requirements for commercial or Phase III clinical use in combination with GMP protocols requiring products to be infused fresh, shortly after processing, result in the design of clinical laboratories that are not cost-effective.

## Chemokine secretion and therapeutic outcome

ISCT stablished guidelines for the phenotypical identification of MSCs however, phenotype does not necessarily equate with therapeutic potential. The mechanism behind the tissue-building and anti-inflammatory role of MSCs is not fully understood but are partially mediated by chemokine secretion. However, there are few studies aimed at understanding the differences between MSCs isolated from different sources as BM MSCs are the main source of MSCs used in the clinic.

Wharton's jelly (WJ) of the umbilical cord holds huge potential as an MSC source, and comparative studies have shown that CXCL1, CXCL2, CXCL5, CXCL6 and CXCL8, strong angiogenic chemokines, are upregulated in WJ MSC compared with BM MSC. In contrast, CXCL12 and CXCL13, which are also potent angiogenic factors, were found to be upregulated in BM MSCs [87].

Transcriptional profiling of identically cultured umbilical cord (UC) derived MSCs and adipose derived MSCs showed that these MSCs differed widely in the expression of anti-inflammatory and angiogenic genes in response to inflammatory stimulation [13]. CXCL1, CXCL2, CXCL3, CXCL5, CXCL6 and CXCL8 were dramatically upregulated in UC MSCs compared with Ad MSCs, while the angiostatic chemokines CXCL10, CXCL9 and CXCL14 were upregulated in Ad MSCs. CCL20 was the only inflammatory chemokine transcribed at higher rate by UC MSCs, while CCL1, CCL3, CCL4, CCL5, CCL7, CCL11, CCL13, and CX3CL1 were transcribed at the highest rates by Ad MSCs.

Differential chemokine transcription levels resulted in a differential therapeutic potential in a diabetic mouse model, where islet co-transplantation with UC MSCs into diabetic mice resulted in a better glycemic regulation compared to islet co-transplantation with identically cultured Ad MSCs [13].

Following a similar standardized approach, low purity islet (LPI) derived MSCs and BM MSCs were compared regarding their immunomodulatory, pro-angiogenic and chemotactic potential and it was observed that they expressed similar transcriptional patterns of chemoattractant and inflammation-modulating molecules, with the exception of CX3CL1, which was transcribed at marginally higher levels by LPI MSCs. Comparison of the immunoregulatory potential of LPI and BM MSCs resulted in identical T cell suppression potential in vitro and no significant differences in the total number of immune cells migrating towards the MSCs when infused into an in vivo migration model [12].

## Concluding remarks

**Fig. 2:**
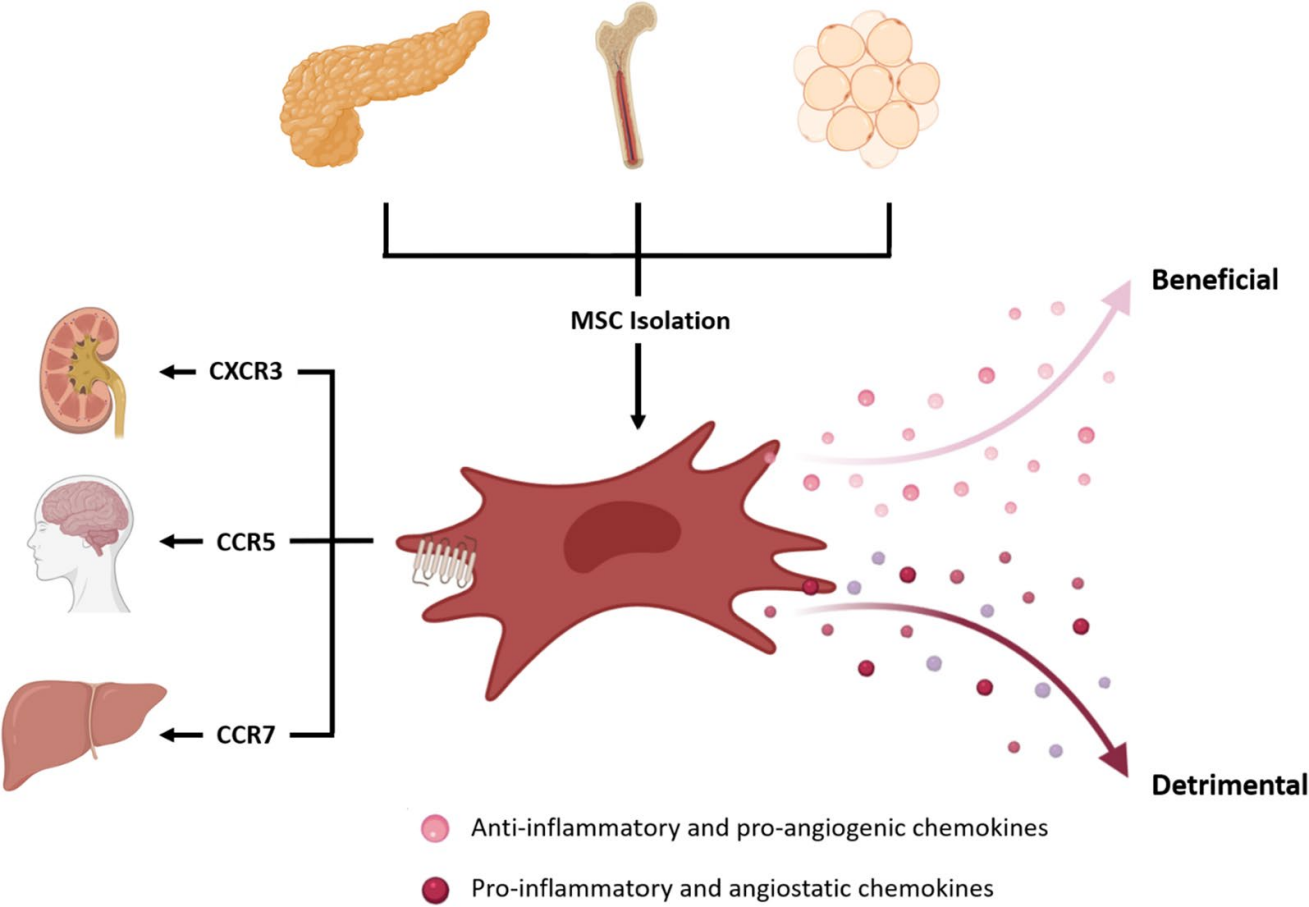
Chemokine receptor expression and chemokine secretion predict therapeutic potential. MSCs isolated from different tissues have a differential chemokine receptor expression and chemokine secretion that results in differential potential as cellular therapeutics

The recruitment and homing of MSCs to sites of injury is essential to contribute to tissue repair, revascularization and regeneration, as well as to dampen inflammation and avoid the activation of the immune system. MSCs isolated from different tissues express different chemokine receptors and thus, tissue source of isolation could dictate MSCs migration potential (Fig. 2). Understanding the role of specific chemokine receptors in relation to migration towards specific anatomical locations could make MSCs isolated from some tissue sources more desirable than others for specific clinical settings, including autoimmune diseases like psoriasis and diabetes, transplantation, or acute injuries.

Analysis of chemokines at transcription and protein levels have shown that anti-inflammatory and pro-angiogenic phenotypes correlated with positive outcomes in a transplant model [13]. Chemokine profiling enables prediction of leukocyte recruitment, immunomodulatory potential and T cell inhibition potential and thus, the therapeutic outcome [12] (Fig. 2). BM MSCs are the most used MSCs within the clinic but their frequency within the tissue is very low and their isolation represents a burden for the donor. Screening of anti-inflammatory and pro-angiogenic genes represents a new approach for identification of alternative MSC sources suitable for therapy and broadens the choices for MSC manufacturing. Thus, we conclude that chemokine profiling can be used to predict the in vivo therapeutic potential of MSCs isolated from new sources and to compare them to the well-known BM MSCs.

## Data Availability

Data sharing is not applicable to this article as no datasets were generated or analysed during the current study.

## Abbreviations

ACKR: Atypical chemokine receptor
Ad: Adipose
BM: Bone marrow
CFU-F: Colony-forming unit fibroblast
GMP: Good Manufacturing practice
HGF: Hepatocyte growth factor
IDO: Indoleamine 2, 3-dioxgenase
IL-10: Interleukin 10
ISCT: International Society of Cellular Therapy
LPI: Low purity islet
MMP9: Matrix metalloproteinase
MSC: Mesenchymal stromal cell
TGF-β: Transforming growth factor beta
TSG6: Tumor necrosis factor-inducible gene 6 protein
UC: Umbilical cord
VEGF: Vascular endothelial growth factor
